# Microbial Metabolism of the Soy Isoflavones Daidzein and Genistein in Postmenopausal Women: Human Intervention Study Reveals New Metabotypes

**DOI:** 10.3390/nu15102352

**Published:** 2023-05-17

**Authors:** Sebastian T. Soukup, Ann Katrin Engelbert, Bernhard Watzl, Achim Bub, Sabine E. Kulling

**Affiliations:** 1 Department of Safety and Quality of Fruit and Vegetables, Max Rubner-Institut, Federal Research Institute of Nutrition and Food, Haid-und-Neu-Straße 9, 76131 Karlsruhe, Germany; 2 Department of Physiology and Biochemistry of Nutrition, Max Rubner-Institut, Federal Research Institute of Nutrition and Food, Haid-und-Neu-Straße 9, 76131 Karlsruhe, Germany

**Keywords:** isoflavone, daidzein, genistein, human, metabolism, metabotypes

## Abstract

Background: Soy isoflavones belong to the group of phytoestrogens and are associated with beneficial health effects but are also discussed to have adverse effects. Isoflavones are intensively metabolized by the gut microbiota leading to metabolites with altered estrogenic potency. The population is classified into different isoflavone metabotypes based on individual metabolite profiles. So far, this classification was based on the capacity to metabolize daidzein and did not reflect genistein metabolism. We investigated the microbial metabolite profile of isoflavones considering daidzein and genistein. Methods: Isoflavones and metabolites were quantified in the urine of postmenopausal women receiving a soy isoflavone extract for 12 weeks. Based on these data, women were clustered in different isoflavone metabotypes. Further, the estrogenic potency of these metabotypes was estimated. Results: Based on the excreted urinary amounts of isoflavones and metabolites, the metabolite profiles could be calculated, resulting in 5 metabotypes applying a hierarchical cluster analysis. The metabotypes differed in part strongly regarding their metabolite profile and their estimated estrogenic potency.

## 1. Introduction

Soy isoflavones (IF) are plant secondary metabolites, and they belong to the group of phytoestrogens due to their ability to interact with the intracellular estrogen receptor (ER) α and ER β [1,2]. Due to their estrogenic potency and further biological activities, IF are associated with beneficial health effects such as the alleviation of menopausal symptoms and the prevention of osteoporosis [3]. However, IF are also discussed to have adverse effects, especially in specific risk groups [4]. In this regard, IF might have an impact on breast cancer by proliferative effects on existing ER-sensitive breast cancer cells and might influence the thyroid hormone system [4].

**Figure 1:**
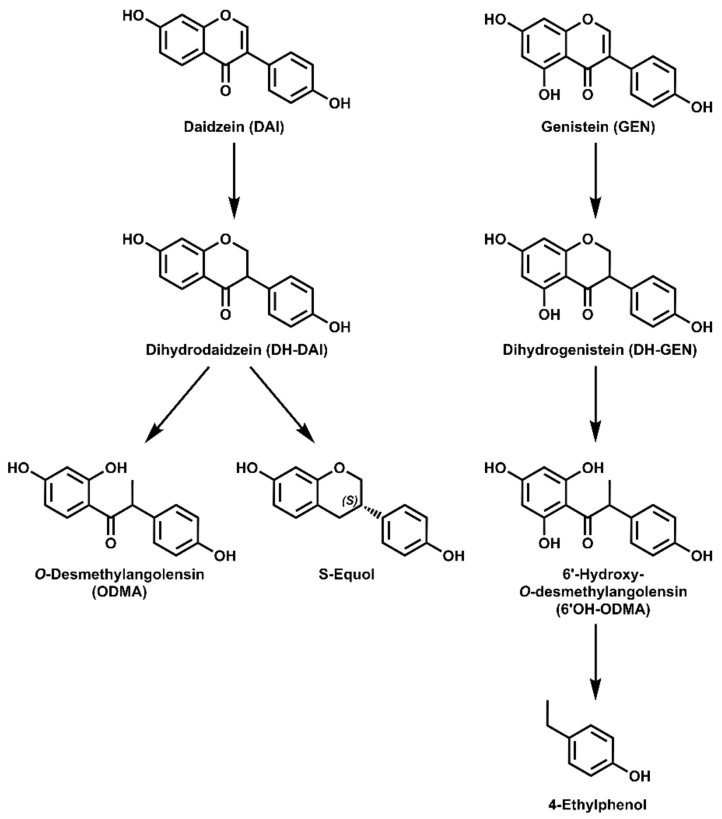
Potential metabolites of daidzein (DAI) and genistein (GEN) formed by human gut microbiota.

Daidzein (DAI), genistein (GEN), and, to a lesser proportion, glycitein are the main IF in soy. IF undergo a complex microbial metabolism in the gut. DAI can be converted to dihydrodaidzein (DH-DAI), followed by the formation of *O*-desmethylangolensin (ODMA) or S-equol. GEN can be metabolized analogously to dihydrogenistein (DH-GEN) and can be further transformed to 6′-hydroxy-*O*-desmethylangolensin (6′OH-ODMA) and 4-ethylphenol (4EP) [4]. These possible metabolic pathways of DAI and GEN are illustrated in Figure 1.

However, the gut microbiota is not capable of producing each of these metabolites in every individual. As a consequence, this causes interindividual (and interspecies) differences in the resulting metabolite profiles after IF intake. This is due to many factors influencing the composition of the gut microbiota, such as age, sex, genetics, and nutrition [2,4]. Many gut microorganisms are known to degrade IF, including bacteria of the family *Clostridiaceae*, *Eubacteriaceae*, *Eggerthellaceae*, and *Coriobacteriaceae* [5,6,7,8,9].

The inter-individual variation in microbial metabolism led early on to a classification of humans in different IF metabotypes. The distinction primarily concerned the ability to form equol, which led to a division of IF consumers into so-called “equol-producers” and “equol-non-producers” [4,10]. The microbiota of about one-third of the Western population is capable of producing equol from daidzein, whereas the proportion of equol-producer in Asian countries is much higher (50–60%) [11]. A further, more recent grouping of the population is into “ODMA-producers” versus “ODMA-non-producers”, whereby 10–40% of individuals count as “ODMA-non-producers” [11,12]. Both of these IF metabotypes refer only to the degradation of DAI and do not consider the metabolization of GEN. To the best of our knowledge, other IF metabotypes, such as the two mentioned above, are not described yet.

The microbial metabolism of IF has an immense impact on their estrogenic potency, and therefore, the individual metabolite profile following IF ingestion may lead to different individual biological effects. Various in vitro and in vivo systems are available for the study of estrogenic activity, with advantages and limitations. These include the ligand-binding assay (LBA), which measures the binding affinity of compounds to ER, and the transactivation assay (TAA) in yeast or mammalian cells, which detects ER-mediated activation of a reporter gene [13]. When comparing the parent compounds, GEN exhibited a higher estrogenic activity than DAI [14,15,16]. The metabolization of DAI to equol lead to a relatively strong increase in estrogenic activity [15,16]. In contrast, degradation of GEN to DH-GEN, 6′OH-ODMA, and/or 4EP results in a decrease in estrogenic potential [15]. Regarding the further metabolites of DAI, ODMA was reported to have similar or, in most cases, higher activity in TAA [15,16,17], whereas DH-DAI exhibited a lower estrogenic potency [15]. With the exception of 4EP and 6′OH-ODMA, DAI and GEN and their metabolites showed higher affinity for ER β than for ER α [15]. 4EP exhibited no detectable ER binding affinity, and 6′OH-ODMA showed comparable values for ER α and ER β [15].

Regarding the capability to metabolize ingested compounds, the metabotyping of the population is not unique to IF and is an important aspect of many polyphenols, especially for understanding the outcomes in studies investigating the effects of these compounds [10,11,18,19,20].

The previous classification of humans into “equol-producers” and “non-equol-producers” or “ODMA-producers” and “ODMA-non-producers” does not reflect the full complex microbial metabolism of IF; in particular, GEN has not been considered. We, therefore, investigated the overall microbial metabolite profile of IF, including the less considered metabolite 4EP, in postmenopausal women, as they are the most relevant target group for the beneficial health effects of IF (menopausal symptoms and osteoporosis) but also represent a potential risk group for breast cancer. Based on the determined microbial metabolite profiles, we derived IF metabotypes and discussed their impact on the estrogenic activity of ingested soy IF.

## 2. Materials and Methods

### 2.1. Chemicals and Reagents

Daidzein (DAI) and genistein (GEN) were purchased from LC Laboratories (Woburn, MA, USA) and had a purity of >99%. Dihydrodaidzein (DH-DAI) (>99%) was purchased from Toronto Research Chemicals (North York, ON, Canada). Dihydrogenistein (DH-GEN) (>99%) and equol (>97%) were purchased from APIN Chemicals Ltd. (Abingdon, UK). *O*-Desmethylangolensin (ODMA) (>99%) and 6′-hydroxy-*O*-desmethylangolensin (6′OH-ODMA) (>99%) were purchased from Plantech UK (Reading, UK). 4-Ethylphenol (4EP) (99%) and [2,3,5,6-D4-4OD]4-Ethylphenol (98 Atom%) were purchased from Sigma-Aldrich (Steinheim, Germany) and Campro Scientific (Berlin, Germany), respectively. [^13^C_3_]Daidzein (^13^C_3_-DAI), [^13^C_3_]genistein (^13^C_3_-GEN), and [^13^C_3_]-*O*-desmethylangolensin (^13^C_3_-ODMA) were provided by Nigel Botting and Nawaf Al-Maharik (University of St. Andrews, UK).

All other chemicals and solvents used were of analytical grade.

### 2.2. Subjects and Study Design

The double-blind, placebo-controlled, randomized trial in parallel design with 179 postmenopausal women has been described previously [21]. The present study refers to a subgroup of women (*n* = 60) who received for 12 weeks a commercially available, analytically well-characterized soy IF extract (for details, see [21], Supplementary Material Table S1) in capsule form for 12 weeks. Daily intake was 117.4 mg IF (genistein 49.7%; daidzein 41.4%; glycitein 9.0%). At the end of the 12-week intervention, the women of this subgroup collected their urine in one day during the wake phase from the morning until evening/night. The individual wake phases of the women led to the different urine collection times (mean collection time: 15.9 h; min–max: 11.4–18.5 h). For each participant, the total volume of the collected urine was determined, and then the urine samples were stored at −80 °C until further processing. One woman was excluded from the study because she had lost significant weight during the study. Thus, 59 women were included in the study, and the data presented refer to these 59 women.

### 2.3. Determination of Microbial Metabolite Profile of IF in Enzymatic Hydrolyzed Urine

Urine samples were prepared as previously described with minor modifications regarding the internal standard mixture [22]. Briefly, 200 µL water-diluted urine (5-fold for equol analysis and 50-fold for analysis of DAI, GEN, DH-DAI, DH-GEN, ODMA, and 6′OH-ODMA) were mixed with 5 µL of internal standard mixture (^13^C_3_-DAI, ^13^C_3_-GEN, ^13^C_3_-ODMA, (each 5 µM), and D4-equol (50 µM) in dimethyl sulfoxide (DMSO)). Further sample preparation (incubation with β-glucuronidase and arylsulfatase, liquid-liquid extraction of analytes), as well as LC-MS analysis, were performed as described previously [22]. The injection volume was 20 µL for equol analysis and 20 µL for analysis of the other analytes.

For quantification, an external calibration curve was prepared in blank urine by adding different concentrated analyte standard mixtures at the beginning of sample preparation. The limit of quantitation (LOQ) for each analyte in hydrolyzed urine were as follows: DAI 5.6 fmol, GEN 10.0 fmol, DH-DAI 3.3 fmol, DH-GEN 6.4 fmol, ODMA 3.3 fmol, 6′OH-ODMA 9.4 fmol, and equol 6.8 pmol. We defined the limit of detection (LOD) as one-third of the respective LOQ. For further statistical analyses, values between LOQ and LOD (traces) were set as LOQ/2, and no detected analytes were set as zero.

For validation of the method, the accuracies, precisions, recoveries, and matrix effects were determined for each analyte. Validation results for DAI and equol are described previously [22]. The results of validation for GEN, DH-DAI, DH-GEN, ODMA, and 6′OH-ODMA are summarized in Supplementary Material Table S1.

### 2.4. Determination of 4EP in Urine

5 µL DMSO and 5 µL internal standard (D4-4EP in DMSO) were pipetted in a 10 mL headspace vial. In the case of calibration samples and quality control (QC) samples, 5 µL of respective 4EP standard mixture in DMSO was added instead of 5 µL DMSO. Then 60 µL of study urine samples or blank urine, as well as 150 µL ammonium acetate buffer (0.1 M, pH 5.0) containing 1,4-dithiothreitol (10 mM), 360 U β-glucuronidase (bovine liver, Typ B-3), and 36 U arylsulfatase (Helix pomatia, Typ H-1) were added. The mixtures were incubated for 2 h at 37 °C with gentle shaking. To minimize enzymatic activity after incubation, samples were placed on ice for 2 min. Then the samples were carefully mixed with 210 µL of ice-cold ethanol, which was added through the septum with a syringe. Subsequently, the samples were analyzed by headspace solid-phase microextraction-GC-MS (HS-SPME-GC-MS).

HS-SPME-GC-MS analyses were carried out on a Shimadzu GCMS QP2010 Ultra equipped with a Combi PAL Autosampler. An HS-SPME splitless injection mode was conducted using a Supelco 30/50 µm DVB/CAR-PDMS fiber and the program listed in Table 1. A Phenomenex Zebron ZB-Wax plus column (60 m × 0.25 mm × 0.25 µm) with helium as carrier gas was used. The oven temperature program was as follows: (1) 50 °C for 2 min; (2) 50 °C to 230 °C with 10 °C/min; (3) 230 °C for 10 min, which resulted in a total run time of 30 min. The transfer line was adjusted to 250 °C. The quadrupole MS was operated with a scan range of m/z 35–250. The ion source temperature was set at 200 °C, and 70 eV EI spectra were recorded. For the acquisition, the Scan/SIM (selected ion monitoring)-mode was chosen using m/z 107 (100%) and m/z 122 (60%) for 4EP identification and m/z 111 (100%) and m/z 126 (40%) for D4-4EP (internal standard) identification. For each analyte, the most intense fragment was used for quantification.

For quantification, an external calibration curve was prepared in blank urine by adding different concentrated analyte standard solutions at the beginning of sample preparation (see above). The best-fit line was obtained by linear regression with a weighting of 1/x. The LOQ for 4EP was 400 nM in urine. For further statistical analyses, values below LOQ were set as zero.

To validate the method, QC samples were prepared on each measurement day. For this purpose, a 4EP solution (final concentration in urine: 2 µM 4EP) was added to blank urine before sample preparation (see above). The accuracy and precision for 4EP in the QC samples (in total, 9 QC samples over 5 days) were 90% and 10.8%, respectively.

### 2.5. Data Analysis and Statistics

For each participant, the excreted amounts of analytes in the urine collection period (in µmol) were calculated. For this purpose, the individual concentrations of analytes in the urine were multiplied by the respective urine volume excreted during the urine collection period.

Total excreted amounts of IF and their metabolites were expressed as IF equivalents. For this purpose, the urinary excreted amounts of the DAI, GEN, and each measured metabolite were first calculated in µmol and then summed for each woman.

Hierarchical cluster analysis was conducted to obtain groups with a similar metabolite profile forming a metabotype. For this analysis, the relative excreted amounts of analytes were used, which were calculated for each participant by dividing the individually excreted amount of an analyte by the corresponding IF equivalent. The hierarchical cluster analysis according to the Ward method was carried out with the software JMP 16.2.

## 3. Results

### 3.1. DAI, GEN, and Their Microbial Metabolites in Urine

DAI, GEN, and their microbial metabolites were quantified in the human urine samples by LC-MS and HS-SPME-GC-MS. Based on these results, the excreted amounts of IF and metabolites during the urine collection period were calculated according to Section 2.5. The results of the excreted amounts are summarized in Table 2.

The mean renal excretion of DAI and GEN were 87.2 ± 39.0 μmol and 30.9 ± 23.5 µmol, respectively. The dihydro-metabolites DH-DAI and DH-GEN were detected in 97% and 80%, respectively, of participants’ urine. Detectable concentrations of ODMA and 6′OH-ODMA in urine were present in 95% and 75%, respectively, of women. Equol was detected in 37% of participants, while 4EP was present in 95%. Total renal excretion of IF (IF equivalents) was 210.5 ± 74.8 µmol (mean ± standard deviation).

### 3.2. Hierarchical Cluster Analysis and IF Metabolite Profiles

To compare the metabolite profiles in the urine of the women, the proportions of the analytes related to the respective IF equivalent (relative excreted amounts) were calculated for each participant (see Section 2.5). The results of this calculation are summarized in Table 3.

**Figure 2:**
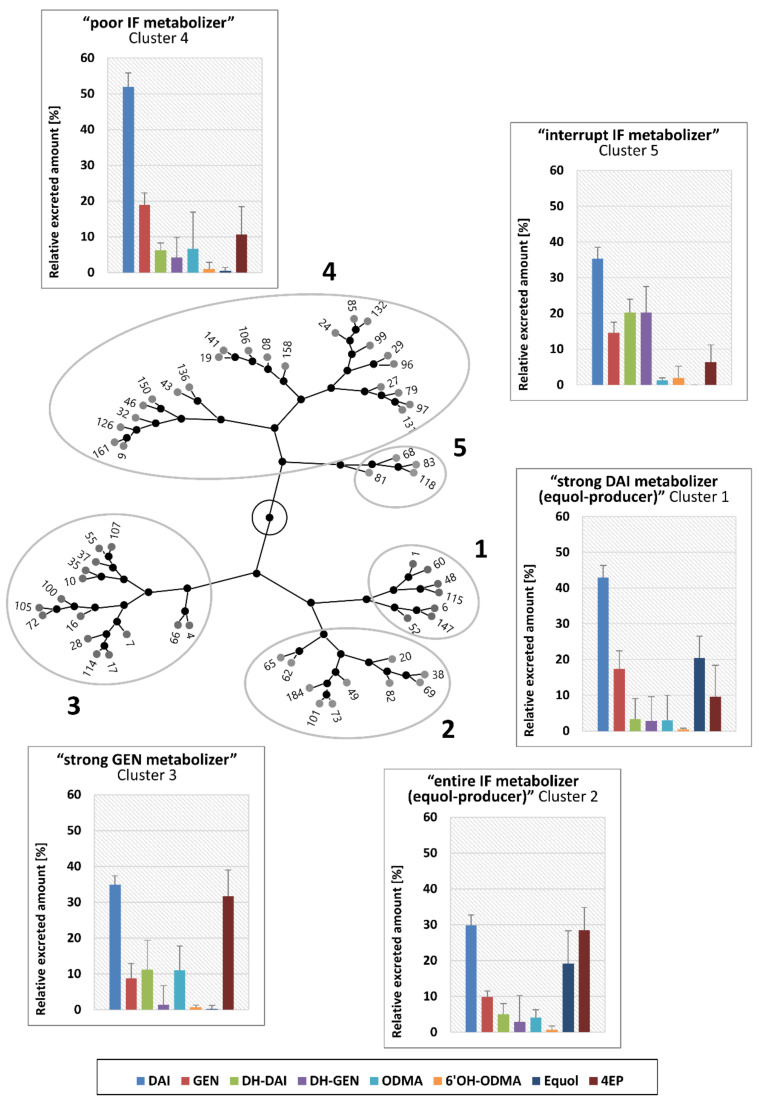
Constellation plot derived from a hierarchical cluster analysis (Ward) of microbial metabolite profiles of isoflavones (IF) measured in the urine of postmenopausal women (*n* = 59), which received soy IF extract for 12 weeks resulting in a per day intake of 117.4 mg IF. Additionally, for each cluster, the metabolite profile (relative excreted amounts of IF and metabolites) is displayed as bar plots. The gray dots with the respective numbers symbolize the individual participants. DAI, daidzein; GEN, genistein; DH, dihydro; ODMA, *O*-desmethylangolensin; 6′OH, 6-hydroxy; 4EP, 4-ethylphenol.

Strong inter-individual differences in the distributions of analytes in the urine samples of women were detected as the pattern of presence or absence of the single metabolites differs between the participants. Therefore, a hierarchical cluster analysis was conducted to identify groups with participants who each exhibited a similar metabolite pattern (“metabotypes”). The results of the hierarchical cluster analysis are displayed as a constellation plot in Figure 2. The cluster membership of individual participants is summarized in Table 3.

Clusters 1 (*n* = 7), 2 (*n* = 10), and 3 (*n* = 15) separate from clusters 4 (*n* = 23) and 5 (*n* = 4). Comparing clusters 1, 2, and 3 with each other, clusters 1 and 2 are most similar to each other, as only these two clusters had high equol proportions (20.4 ± 6.2% and 19.1 ± 9.2%, respectively). The difference between cluster 1 and 2 is due to the higher proportions of 4EP and lower proportion of GEN in cluster 2 compared to cluster 1. In cluster 3, the highest percentage of 4EP overall cluster was detected, in addition to no or only very small (just one member) proportions of equol. Cluster 4, to which the most women were assigned, exhibited the highest proportions of DAI (52.0 ± 6.8%) and GEN (18.9 ± 7.3%). In contrast, cluster 5, with only four women, had strikingly high proportions of the dihydro-metabolites DH-DAI (20.3 ± 6.7%) and DH-GEN (20.2 ± 10.2%), which were 1.8- to 6.0-fold and 4.9- to 13.9-fold higher, respectively, than the mean values of the other four clusters.

The effect of the different metabolization of IF (IF metabotypes) on the estrogenic activity was estimated based on the reported data by Pfitscher et al. [15]. Pfitscher et al. investigated the estrogenic activity of IF and their metabolites in vitro by using an ER (α and β) ligand-binding assay (LBA) as well as an ER α mediated transactivation assay in yeast (TAA). This study was selected because all metabolites included in the metabotyping were investigated, thus avoiding bias due to consulting data from different studies using different test systems or experimental conditions.

To estimate the estrogenic potency of the clusters, the reported metabolite-specific IC50 value and estrogenic potency, respectively, obtained from LBA and TAA, were 1/x transformed in the first step. Then for each cluster, the individual transformed values were multiplied by the proportion of the respective compound, and within the single clusters, these results for each compound were summed up. Finally, the summed estrogenic potencies of the cluster were related to the cluster with the highest value (Table 4).

## 4. Discussion

DAI, GEN, and their microbial metabolites were quantified in the urine samples of 59 postmenopausal women who received soy IF extract for 12 weeks. Based on these results, the excreted amounts of IF and metabolites were calculated, and a hierarchical cluster analysis was conducted to identify clusters that each exhibited a similar metabolite pattern (metabotypes). The five clusters obtained, and their metabolite profiles are discussed in the following.

The first main separation in clusters 1, 2, and 3 on the one side and clusters 4 and 5 on the other side seem to be based on the higher metabolizing capability of the gut microbiota of women in clusters 1, 2, and 3 to transform DAI to equol and/or GEN to 4EP. In contrast, the gut microbiota of women in clusters 4 and 5 exhibited correspondingly lower metabolizing activity, as reflected by the highest proportions of DAI and GEN in cluster 4. In this context, cluster 5 seems to be very specific to which only four women could be assigned: The gut microbiota showed a higher degradation activity compared to cluster 4, but the IF metabolism seems to “stop” at the step of the dihydro-metabolites DH-DAI and DH-GEN. It is known that different microorganisms are responsible for the single steps of the IF metabolic pathway [9,23]. So, the four women of cluster 5 seem to have no or lower amounts of microorganisms capable of further metabolizing DH-DAI and DH-GEN.

Women assigned to clusters 1 and 2 belong to the group of so-called equol-producers, as their gut microbiota is able to metabolize daidzein to equol. However, these two clusters differ from each other with regard to the microbial degradation of GEN to 4EP. In detail, the gut microbiota of women in cluster 1 seems to metabolize GEN only to a lesser extent, as the proportion of GEN was markedly higher in addition to the 3-fold lower proportion of 4EP. Regarding the metabolization of DAI to equol and GEN to 4EP, cluster 3 seems to be the opposite of cluster 1. This is due to the fact that in cluster 3, besides the highest proportion of 4EP in all clusters, no or only very low (only in a single woman) proportions of equol were detected. It can be concluded that the gut microbiota of women in cluster 3 appears to have a relatively high capability to metabolize GEN to 4EP but no or very low amounts of equol-forming bacteria.

As presented, the microbial metabolism of IF and the resulting metabolite profiles are complex, and, with the exception of equol, a designation based on the production or non-production of a specific metabolite does not consider this complexity. Therefore, we propose the following naming of the clusters: “strong DAI metabolizer (equol-producer)” for cluster 1, “entire IF metabolizer (equol-producer)” for cluster 2, “strong GEN metabolizer” for cluster 3, “poor IF metabolizer” for cluster 4, and “interrupt IF metabolizer” for cluster 5.

Regarding the estimation of the cluster’s estrogenic potency (Table 4), cluster 1 (“strong DAI metabolizer (equol-producer)”) exhibited—in all three assays—the highest one due to strong metabolization of DAI to equol and weak degradation of GEN. In addition, in all assays, cluster 3 (“strong GEN metabolizer”) showed the lowest estrogenic potency (one-third to half of cluster 1), sharing in TAA the last rank with cluster 5 (“interrupt IF metabolizer”). This is because in both clusters, no equol is formed, and GEN is strongly degraded to 4EP or DH-GEN. In terms of affinity to ER α and ER β, clusters 2, 4, and 5, which showed more or less similar activities, ranked second. However, based on data of the ER α mediated TAA, cluster 2 (“entire IF metabolizer (equol-producer)”) alone has the second highest activity, which is 0.82-fold the potency of cluster 1. This is driven by the higher estrogenic potency of equol in TAA compared to LBA. Cluster 4 (“poor IF metabolizer”) exhibited a medium potency compared to cluster 1 due to the preservation of DAI and especially GEN. In summary, the different metabotypes (cluster) showed, in part, relatively strong differences regarding estrogenic activities, which might have an influence on the effects of ingested IF. It must be noted that this is an estimate of the estrogenic potencies of the different IF metabotypes based on in vitro data. Results may vary when data from other assays are used. However, the assessment gives an impression of the impact of the metabotype on the estrogenic potency of ingested soy IF.

Regarding the impact of the IF metabotypes on health, more aspects must be considered than the estrogenic activity of the metabolites, e.g., pharmacokinetic parameters such as the maximum plasma concentration of metabolites [24]. Furthermore, the different metabotypes are based on different compositions of the gut microbiota, and it is known that the gut microbiota and its composition play a role in human health. This aspect should be considered [25,26,27]. However, the impact of the different IF metabotypes described in the presented study on general health aspects based on the different compositions of gut microbiota was not the focus of this study and should be investigated in further research.

The strength of the presented study is the consideration of the entire known microbial metabolite pathway of IF in humans to derive IF metabotypes. However, it has to be noted that during the 12 weeks of IF intervention, full control of the participants’ diet was not possible. Regarding 4EP, it cannot be ruled out that parts of the detected amounts could come from food sources other than the ingested soy extract. It has been described that 4EP can also be a microbial metabolite of quercetin, which is a phytochemical in some foods such as apples and onions [28]. However, we are confident that relevant amounts of the detected 4EP arise from the ingested IF due to the daily relatively high dose of IF intake over a long time period as well as further observations that a single intake of approx. half a dose of the same soy extract led to relatively high 4EP urinary concentrations in postmenopausal women. Therefore, excluding 4EP from the cluster analyses would not consider the whole relevant microbial metabolite profile of IF in the presented study.

In conclusion, the presented study postulates new IF metabotypes that have different estrogenic potencies. These metabotypes need to be confirmed in further human studies with a larger number of participants and, therefore, should be determined in human studies addressing the effects of IF. In addition to the metabotypes presented, further aspects have to be considered when assessing the effects of IF in humans, such as their bioavailability, the distribution in the body, and further phase II metabolism. These aspects were not the focus of the presented study, and other experiments have to be conducted to address them. However, the presented results provide further insight into the inter-individual metabolism of IF and its impact on estrogenic activity and may help to understand the outcomes of human studies on the effects of IF.

## 5. Conclusions

Our data reveal further insight into the variability of the inter-individual metabolism of isoflavones, indicating novel metabotypes of isoflavone metabolism. This information can help to better understand the outcomes of human studies on the biological effects of soy isoflavones.

## Figures and Tables

**Table 1:** HS-SPME program used in the HS-SPME-GC-MS analysis of 4-ethylphenol (4EP) in urine samples.

Parameter	Setting
Pre-Injection Time (s)	600
Incubation Temp (°C)	80
Agitator speed (rpm)	250
Agitator on time (s)	9
Agitator off time (s)	1
Needle Penetration (mm)	22
Fiber Penetration (mm)	22
Extraction time (s)	1800
Injector Penetration (mm)	32
Desorption time (s)	90
Post Fiber Conditioning (s)	1800

**Table 2:** Excreted amounts [µmol] of isoflavones (IF) and their microbial metabolites in the urine collection period (mean collection time: 15.9 h; min–max: 11.4–18.5 h) at the end of the intervention study. The postmenopausal women consumed 117.4 mg IF daily for 12 weeks with soy IF extract. DAI, daidzein; GEN, genistein; DH, dihydro; ODMA, *O*-desmethylangolensin; 6′OH, 6-hydroxy; 4EP, 4-ethylphenol; SD, standard deviation.

Participant No.	DAI	GEN	DH-DAI	DH-GEN	ODMA	6′OH-ODMA	Equol	4EP	IF Equivalents
1	110.5	52.2	14.0	13.6	4.0	0.7	31.7	7.2	233.9
4	42.7	10.5	48.4	10.9	4.7	0.2	0.0	43.0	160.2
6	39.8	15.4	2.6	0.6	4.2	0.4	21.0	1.1	85.1
7	60.4	12.1	8.7	0.0	24.2	0.0	0.0	88.2	193.5
9	127.2	63.9	9.9	29.7	0.0	0.0	0.0	0.0	230.7
10	60.5	21.6	26.3	8.8	18.0	2.4	0.0	42.6	180.3
16	107.0	32.3	5.2	0.9	22.4	2.8	0.0	66.1	236.6
17	92.6	13.6	22.9	4.6	24.4	0.0	0.0	100.3	258.3
19	94.1	26.4	0.2	1.1	22.5	1.2	0.0	26.5	172.0
20	60.8	23.2	22.6	17.8	2.1	9.0	42.9	60.9	239.3
24	82.7	21.0	22.7	1.2	16.1	0.6	0.0	43.8	188.0
27	64.0	23.5	4.6	3.1	3.4	1.1	3.0	22.2	124.9
28	81.8	19.4	42.1	1.5	19.2	1.6	0.0	78.7	244.3
29	73.3	16.5	12.3	0.0	20.2	0.9	0.0	13.7	136.8
32	91.3	50.4	14.4	41.0	0.3	0.0	0.0	7.3	204.7
35	60.4	18.1	29.1	3.8	26.5	0.6	0.0	30.7	169.1
37	55.3	9.6	11.9	0.9	26.1	0.9	6.2	48.9	159.8
38	71.3	25.8	26.2	5.2	17.9	0.8	20.6	49.9	217.7
43	128.5	39.1	0.0	0.0	6.7	0.8	0.0	2.4	177.5
46	104.2	67.5	22.5	11.0	1.7	0.0	0.0	2.9	209.8
48	124.9	59.7	3.7	4.2	2.3	0.6	36.4	70.9	302.8
49	34.5	10.5	3.2	3.2	8.6	0.0	25.1	57.4	142.5
52	60.9	22.6	2.1	0.0	4.1	1.1	43.0	15.7	149.4
55	63.3	14.6	19.5	0.0	21.6	3.3	0.0	40.4	162.7
60	60.3	26.2	6.5	16.6	0.9	1.6	30.1	18.1	160.3
62	59.7	19.8	1.2	0.7	5.7	0.4	67.8	49.2	204.3
65	42.2	19.3	7.9	17.2	0.7	0.7	69.6	36.7	194.3
66	64.6	16.0	66.6	7.3	24.3	0.8	0.0	124.2	303.7
68	187.2	55.5	137.1	53.8	4.1	33.5	0.0	12.6	483.7
69	71.0	15.8	16.9	2.0	11.4	0.3	16.6	48.3	182.4
72	64.3	20.5	0.6	0.0	25.4	0.3	0.0	65.3	176.4
73	67.7	17.9	4.2	1.1	7.7	0.7	39.6	66.2	205.1
79	52.3	20.6	1.9	1.6	0.4	0.0	1.1	14.5	92.3
80	147.0	36.3	16.1	5.2	22.5	3.0	0.0	53.8	283.8
81	38.7	22.3	34.9	53.1	1.3	0.0	0.0	3.1	153.4
82	91.6	36.0	21.1	18.0	4.4	1.2	31.4	76.3	279.9
83	67.4	26.7	27.7	25.4	3.3	0.0	0.0	14.2	164.9
85	84.0	28.8	29.9	2.5	8.6	0.5	0.0	33.1	187.4
96	107.6	32.8	31.0	3.7	15.8	1.0	0.0	11.8	203.7
97	134.1	48.8	4.8	9.2	4.9	5.1	7.3	29.9	244.1
99	59.2	17.8	18.8	5.1	1.1	10.6	0.0	13.0	125.6
100	93.0	25.7	13.5	0.0	22.4	0.0	0.0	82.0	236.6
101	47.7	18.5	3.6	0.5	4.9	0.3	36.6	52.7	164.9
105	107.4	34.0	4.8	0.4	37.6	1.1	0.0	84.0	269.4
106	65.8	13.3	5.6	0.0	22.6	0.7	0.0	22.7	130.6
107	66.5	15.2	24.4	0.9	28.1	3.7	0.0	52.5	191.3
114	90.6	14.9	27.8	3.5	18.1	0.8	0.0	88.3	244.0
115	91.7	35.7	2.2	1.9	2.6	0.4	42.7	38.4	215.7
118	108.0	47.4	38.8	58.6	5.2	2.1	0.0	35.9	296.0
126	226.9	130.6	23.4	69.2	0.0	0.0	0.0	0.0	450.1
131	122.9	43.0	10.3	6.1	6.3	2.2	7.2	29.1	227.2
132	97.8	30.4	36.5	13.0	13.1	11.6	0.0	43.1	245.4
136	64.2	18.7	0.1	0.0	19.4	0.0	0.0	2.1	104.4
141	124.3	27.8	0.0	0.0	37.8	0.9	0.0	36.3	227.1
147	78.8	24.0	12.4	1.3	16.6	0.3	44.3	0.0	177.8
150	216.1	131.1	21.6	9.2	1.7	0.0	0.0	12.4	392.1
158	78.4	15.4	5.6	0.0	45.3	0.0	0.0	38.9	183.6
161	88.1	46.2	7.0	22.6	0.0	0.0	0.0	0.2	164.1
184	86.8	22.7	2.5	0.0	24.0	1.6	42.8	96.2	276.7
**Min**	**34.5**	**9.6**	**0.0**	**0.0**	**0.0**	**0.0**	**0.0**	**0.0**	**85.1**
**Max**	**226.9**	**131.1**	**137.1**	**69.2**	**45.3**	**33.5**	**69.6**	**124.2**	**483.7**
**Mean**	**87.2**	**30.9**	**17.7**	**9.7**	**12.7**	**1.9**	**11.3**	**39.1**	**210.5**
**SD**	**39.0**	**23.5**	**21.1**	**15.8**	**11.2**	**4.8**	**18.7**	**30.1**	**74.8**

**Table 3:** Relative excreted amounts of isoflavones (IF) and metabolites [%] (related to the respective IF equivalent) during the urine collection period at the end of the intervention study (mean collection time: 15.9 h; min–max: 11.4–18.5 h) of postmenopausal women, which received soy IF extract for 12 weeks resulting in a per day intake of 117.4 mg IF. DAI, daidzein; GEN, genistein; DH, dihydro; ODMA, *O*-desmethylangolensin; 6′OH, 6-hydroxy; 4EP, 4-ethylphenol; SD, standard deviation.

Participant No.	DAI	GEN	DH-DAI	DH-GEN	ODMA	6′OH-ODMA	Equol	4EP	Belonging to Cluster
1	47.2	22.3	6.0	5.8	1.7	0.3	13.6	3.1	1
6	46.7	18.1	3.1	0.7	4.9	0.5	24.7	1.3	1
48	41.3	19.7	1.2	1.4	0.8	0.2	12.0	23.4	1
52	40.7	15.1	1.4	0.0	2.7	0.8	28.8	10.5	1
60	37.6	16.3	4.0	10.4	0.6	1.0	18.8	11.3	1
115	42.5	16.6	1.0	0.9	1.2	0.2	19.8	17.8	1
147	44.3	13.5	7.0	0.7	9.4	0.2	24.9	0.0	1
**Mean**	**42.9**	**17.4**	**3.4**	**2.8**	**3.0**	**0.5**	**20.4**	**9.6**	
**SD**	**3.4**	**2.9**	**2.4**	**3.9**	**3.2**	**0.3**	**6.2**	**8.8**	
20	25.4	9.7	9.4	7.4	0.9	3.8	17.9	25.4	2
38	32.7	11.8	12.1	2.4	8.2	0.4	9.5	22.9	2
49	24.2	7.4	2.2	2.2	6.0	0.0	17.6	40.3	2
62	29.2	9.7	0.6	0.3	2.8	0.2	33.2	24.1	2
65	21.7	9.9	4.1	8.9	0.4	0.4	35.8	18.9	2
69	38.9	8.7	9.3	1.1	6.2	0.1	9.1	26.5	2
73	33.0	8.8	2.1	0.5	3.7	0.4	19.3	32.3	2
82	32.7	12.8	7.5	6.4	1.6	0.4	11.2	27.3	2
101	28.9	11.2	2.2	0.3	3.0	0.2	22.2	32.0	2
184	31.4	8.2	0.9	0.0	8.7	0.6	15.5	34.8	2
**Mean**	**29.8**	**9.8**	**5.0**	**3.0**	**4.2**	**0.7**	**19.1**	**28.5**	
**SD**	**5.1**	**1.7**	**4.2**	**3.3**	**3.0**	**1.1**	**9.2**	**6.4**	
4	26.7	6.5	30.2	6.8	2.9	0.1	0.0	26.8	3
7	31.2	6.2	4.5	0.0	12.5	0.0	0.0	45.6	3
10	33.6	12.0	14.6	4.9	10.0	1.3	0.0	23.6	3
16	45.2	13.6	2.2	0.4	9.5	1.2	0.0	27.9	3
17	35.8	5.2	8.9	1.8	9.5	0.0	0.0	38.8	3
28	33.5	7.9	17.2	0.6	7.9	0.7	0.0	32.2	3
35	35.7	10.7	17.2	2.3	15.7	0.3	0.0	18.1	3
37	34.6	6.0	7.4	0.6	16.3	0.6	3.9	30.6	3
55	38.9	9.0	12.0	0.0	13.3	2.0	0.0	24.8	3
66	21.3	5.3	21.9	2.4	8.0	0.3	0.0	40.9	3
72	36.4	11.6	0.4	0.0	14.4	0.2	0.0	37.0	3
100	39.3	10.9	5.7	0.0	9.5	0.0	0.0	34.6	3
105	39.9	12.6	1.8	0.1	14.0	0.4	0.0	31.2	3
107	34.8	7.9	12.8	0.5	14.7	1.9	0.0	27.5	3
114	37.1	6.1	11.4	1.4	7.4	0.3	0.0	36.2	3
**Mean**	**34.9**	**8.8**	**11.2**	**1.5**	**11.0**	**0.6**	**0.3**	**31.7**	
**SD**	**5.6**	**2.9**	**8.2**	**2.0**	**3.8**	**0.7**	**1.0**	**7.3**	
9	55.1	27.7	4.3	12.9	0.0	0.0	0.0	0.0	4
19	54.7	15.3	0.1	0.6	13.1	0.7	0.0	15.4	4
24	44.0	11.2	12.1	0.6	8.5	0.3	0.0	23.3	4
27	51.2	18.8	3.7	2.5	2.7	0.9	2.4	17.8	4
29	53.6	12.0	9.0	0.0	14.8	0.6	0.0	10.0	4
32	44.6	24.6	7.1	20.0	0.2	0.0	0.0	3.5	4
43	72.4	22.1	0.0	0.0	3.8	0.5	0.0	1.4	4
46	49.7	32.2	10.7	5.2	0.8	0.0	0.0	1.4	4
79	56.6	22.3	2.0	1.7	0.4	0.0	1.2	15.7	4
80	51.8	12.8	5.7	1.8	7.9	1.0	0.0	18.9	4
85	44.8	15.4	16.0	1.3	4.6	0.2	0.0	17.7	4
96	52.8	16.1	15.2	1.8	7.7	0.5	0.0	5.8	4
97	54.9	20.0	2.0	3.8	2.0	2.1	3.0	12.2	4
99	47.1	14.2	14.9	4.1	0.9	8.5	0.0	10.4	4
106	50.3	10.2	4.3	0.0	17.3	0.5	0.0	17.4	4
126	50.4	29.0	5.2	15.4	0.0	0.0	0.0	0.0	4
131	54.1	18.9	4.6	2.7	2.8	1.0	3.2	12.8	4
132	39.8	12.4	14.9	5.3	5.3	4.7	0.0	17.6	4
136	61.4	17.9	0.1	0.0	18.6	0.0	0.0	2.0	4
141	54.7	12.2	0.0	0.0	16.6	0.4	0.0	16.0	4
150	55.1	33.4	5.5	2.3	0.4	0.0	0.0	3.2	4
158	42.7	8.4	3.1	0.0	24.7	0.0	0.0	21.2	4
161	53.7	28.2	4.3	13.8	0.0	0.0	0.0	0.1	4
**Mean**	**52.0**	**18.9**	**6.3**	**4.2**	**6.7**	**1.0**	**0.4**	**10.6**	
**SD**	**6.8**	**7.3**	**5.3**	**5.7**	**7.3**	**1.9**	**1.0**	**7.8**	
68	38.7	11.5	28.4	11.1	0.8	6.9	0.0	2.6	5
81	25.2	14.6	22.7	34.6	0.8	0.0	0.0	2.0	5
83	40.9	16.2	16.8	15.4	2.0	0.0	0.0	8.6	5
118	36.5	16.0	13.1	19.8	1.8	0.7	0.0	12.1	5
**Mean**	**35.3**	**14.6**	**20.3**	**20.2**	**1.4**	**1.9**	**0.0**	**6.3**	
**SD**	**7.0**	**2.2**	**6.7**	**10.2**	**0.6**	**3.3**	**0.0**	**4.9**	

**Table 4:** Estimated estrogenic potencies of the different isoflavone (IF) metabotypes (clusters) based on IF microbial metabolite profile incorporating the estrogenic activities of the individual metabolites, reported by Pfitscher et al. [15]. For each assay, the estrogenic potencies are related to the cluster with the highest value. ER, estrogen receptor; LBA, ligand-binding assay; TAA, transactivation assay.

Cluster	LBA ER α	LBA ER β	TAA ER α
1	1.00	1.00	1.00
2	0.75	0.75	0.82
3	0.50	0.48	0.27
4	0.81	0.80	0.44
5	0.71	0.70	0.30

## Data Availability

Data is contained within the article or Supplementary Material or available on request.

## Supplementary Materials

The following supporting information can be downloaded at: https://www.mdpi.com/article/10.3390/nu15102352/s1, Table S1: Results of the validation (accuracy, intra-day precision, recovery, and matrix effect) of LC-MS analyses of daidzein, genistein and corresponding microbial metabolites in enzymatic hydrolyzed urine samples.
