# Supplementary material for: Microbial Metabolism of the Soy Isoflavones Daidzein and Genistein in Postmenopausal Women: Human Intervention Study Reveals New Metabotypes
Source: Nutrients. 2023 May 17;15(10):2352. doi: 10.3390/nu15102352 (PMC10223177; doi:10.3390/nu15102352)
Supplement: Supplementary file 1 [file nutrients-15-02352-s001.zip › nutrients-2316337-supplementary.pdf]

Supplementary Material Table S1: Results of the validation (accuracy, intra-day precision, recovery, and matrix effect) of LC-MS analyses of daidzein, genistein and corresponding microbial metabolites in enzymatic hydrolyzed urine samples.

For accuracy and intra-day precision analyte-free urine samples were spiked with a standard mixture of analytes (n=6; final concentration in injected samples 1.25 µM for each analyte) and processed as described. For the validation experiment only $^{13}C_3$-daidzein was added as internal standard.

Additionally, six analyte-free urine samples, which were not spiked with the standard mixture prior to extraction, were processed and spiked during solvation of extraction residue prior to LC-MS analysis. To calculate recoveries, the peak areas of analytes in these samples were compared to peak areas of analytes in urine samples spiked with analytes before extraction. For calculation of matrix effect the peak areas of analytes in urine samples spiked with analytes after extraction and prior to LC-MS analysis were compared to peak areas of analytes with the same concentration (1.25 µM for each analyte) just in solvent without urine matrix.

	**Accuracy [%]**	**Intra-day precision [%]**	**Recovery (mean ± SD) [%]**	**Matrix effect (mean ± SD) [%]**
Genistein (GEN)	114	3.5	99 ± 4	100 ± 2
Dihydrodaidzein (DH-DAI)	100	1.9	102 ± 6	97 ± 4
Dihydrogenistein (DH-GEN)	116	3.1	98 ± 4	99 ± 5
O-Desmethylangolensin (ODMA)	104	3.0	98 ± 2	100 ± 4
6'-hydroxy-*O*-Desmethyl-angolensin (6’OH-ODMA)	101	4.6	98 ± 2	98 ± 3
